# Liver cirrhosis and complications from the perspective of dysbiosis

**DOI:** 10.3389/fmed.2023.1320015

**Published:** 2024-01-16

**Authors:** Guole Nie, Honglong Zhang, Danna Xie, Jun Yan, Xun Li

**Affiliations:** ^1^The First School of Clinical Medicine, Lanzhou University, Lanzhou, China; ^2^Department of General Surgery, The First Hospital of Lanzhou University, Lanzhou, China; ^3^Key Laboratory of Biotherapy and Regenerative Medicine of Gansu Province, Lanzhou, China; ^4^Cancer Prevention and Control Center of Lanzhou University Medical School, Lanzhou, China; ^5^Gansu Institute of Hepatobiliary and Pancreatic Surgery, Lanzhou, China; ^6^Gansu Clinical Medical Research Center of General Surgery, Lanzhou, China

**Keywords:** liver cirrhosis, dysbiosis, complication, portal vein thrombosis, gut-liver axis

## Abstract

The gut-liver axis refers to the intimate relationship and rigorous interaction between the gut and the liver. The intestinal barrier’s integrity is critical for maintaining liver homeostasis. The liver operates as a second firewall in this interaction, limiting the movement of potentially dangerous compounds from the gut and, as a result, contributing in barrier management. An increasing amount of evidence shows that increased intestinal permeability and subsequent bacterial translocation play a role in liver damage development. The major pathogenic causes in cirrhotic individuals include poor intestinal permeability, nutrition, and intestinal flora dysbiosis. Portal hypertension promotes intestinal permeability and bacterial translocation in advanced liver disease, increasing liver damage. Bacterial dysbiosis is closely related to the development of cirrhosis and its related complications. This article describes the potential mechanisms of dysbiosis in liver cirrhosis and related complications, such as spontaneous bacterial peritonitis, hepatorenal syndrome, portal vein thrombosis, hepatic encephalopathy, and hepatocellular carcinoma, using dysbiosis of the intestinal flora as an entry point.

## 1 Introduction

Liver cirrhosis is a primary cause of morbidity and mortality worldwide. The mortality rate of patients with compensated liver cirrhosis increased five-fold and increased 10-fold in patients with decompensated liver cirrhosis compared with the general population (1). Liver cirrhosis is an advanced chronic liver disease caused by various etiologies, including alcohol abuse, obesity, and hepatitis virus infection, and prevalence of cirrhosis is increasing worldwide (2). Although there are many etiologies of liver cirrhosis, alcoholic liver disease (ALD), nonalcoholic fatty liver disease (NAFLD), and viral hepatitis are the prevalent etiologies of chronic liver disease globally (3, 4). Liver cirrhosis is the end-stage pathological manifestation of chronic liver disease charactered by chronic inflammation, hepatic lobe reconstruction, and the formation of pseudolobule and tubercle (5). The bidirectional interactions of the liver and the intestinal microbiota provide a new perspective for the occurrence and development of liver cirrhosis and its complications (6).

Microbes inhabit specific locations of the body, such as the skin, mouth, respiratory tract, gastrointestinal tract, genitourinary system, etc., and the highest density residing in the gastrointestinal tract (7, 8). The exact time of gut microbial colonization is not known, but intestinal microbial colonization does not occur before birth (9). A variety of internal and external factors will affect the intestinal flora after birth, such as dietary, disease and sleep, etc. (10–13). Metagenomics, metatranscriptomic, and metaproteomic of the human intestinal microbiota show that the intestinal microbiota is an important constituent of the organism and plays a crucial role in human health and disease (14). The portal vein is an important channel for the interaction between the liver and the intestinal microbiota. The portal vein can transport intestinal nutrients, bacteria, and microbial products to the liver (15). Under normal physiological conditions, the intestinal microbiota is essential for liver metabolic functions, maturation and maintenance of immune homeostasis (16), and in turn, the liver regulates the intestinal microbiota and metabolic functions mainly through secreting bile acids (BA) (17).

We review clinical studies on cirrhosis and dysbiosis, and then turn to the topic of cirrhosis-related complications and dysbiosis. Dysbiosis may provide new perspectives on cirrhosis and complications.

## 2 Liver cirrhosis and dysbiosis

Liver cirrhosis affects the intestinal microbiota mainly through two aspects: on the one hand, liver cirrhosis can reduce the synthesis of BA, and resulting in the composition and function of the intestinal microbiota (18, 19). On the other hand, liver cirrhosis can cause portal hypertension, gastrointestinal blood stasis, impair intestinal barrier, and gut dysbiosis. Bacteria and metabolites can enter the portal vein and systemic circulation through translocation of the damaged intestinal barrier, causing an inflammatory state in the body and endotoxemia (20), which have different effects on both cirrhosis and cirrhosis-related complications (21).

Bile acids are the major functional components of bile and are generated by a classical and alternative pathway in the liver (22). BA primarily promote the emulsification of fats and aid the absorption of lipid and fat-soluble vitamins (23, 24). BA and the intestinal microbiota can interact with each other, and BA play an important role in regulating the diversity of intestinal flora and small intestine bacterial overgrowth (25). BA can not only directly affect the integrity of the intestinal barrier via regulating the composition of intestinal flora but promoting the synthesis and antimicrobial peptides secretion of the intestinal epithelial cells (26). Moreover, BA regulate metabolic function, energy consumption, and inflammation through interaction with its receptors, such as farnesoid-X-receptor (FXR) and the vitamin D receptor (VDR) as well as the Takeda G-protein coupled BA receptor (TGR5) (27). The activation of FXR can protect the integrity of the intestinal barrier, reduce bacterial translocation and intestinal inflammation (28, 29).

The intestinal microbiota plays a unique role in BA metabolism and homeostasis in the host (19). The metabolites of the intestinal microbiota with a variety of biological functions, also known as postbiotics, such as short-chain fatty acids, secondary BA, choline metabolites, indole derivatives, vitamins, polyamines, lipids, neurotransmitters, neuroactive compounds, and thalamic-pituitary-adrenal axis hormones play an important role in the body (30). ALD, NAFLD, and viral hepatitis are the leading associated causes of liver cirrhosis worldwide, we have mainly summarized the clinical studies related to dysbiosis in these diseases.

In general, the 16S rRNA gene sequencing analysis showed a significant decrease in the levels of Lachnospiraceae and Ruminococcaceae in patients with liver cirrhosis, while the whole-metagenome shotgun sequencing analysis showed that the level of *Faecalibacterium prausnitzii* from the Ruminococcaceae and of *Coprococcus* spp. from the Lachnospiraceae are significantly reduced (31, 32). Dysbiosis of intestinal flora further aggravates liver injury. Studies have shown that the severity of liver injury is closely related to the severity of intestinal flora dysbiosis (33). Changes in the fecal bacterial flora are manifested by changes in the composition of the major Bacteroidetes and Sclerotiniaceae, which produce short-chain fatty acids (SCFA) that are a source of energy for intestinal epithelial cells, and also regulate BA metabolism and induce modulation of the immune function of the intestinal barrier (34). Dysbiosis of the intestinal flora affects changes in intestinal permeability and intestinal metabolites that may be involved in the progression of cirrhosis and its associated complications (35).

### 2.1 ALD and NAFLD

Liver cirrhosis is the common pathologic of the advanced stage of ALD and NAFLD (36). Alcohol and its metabolites can affect the tight junction between intestinal epithelial cells, impair intestinal barrier function, induce bacterial translocation and endotoxemia (37, 38). Gut dysbiosis is closely related to the occurrence and development of NAFLD and ALD, but the mechanism is not clear (39, 40). The intestinal microbiota can promote the development of human NAFLD and ALD into end-stage liver disease (41, 42), indicating that gut dysbiosis may be the common changes of these diseases. A higher abundance of *Enterobacteriaceae* and *Halomonadaceae*, and lower *Lachnospiraceae*, *Ruminococcaceae*, and *Clostridialies XIV* in alcoholic-related liver cirrhosis than non-alcoholic cirrhotics, whereas non-alcoholic steatohepatitis-related liver cirrhosis (NASH) had a higher level of *Porphyromonadaceae*, *Bacterioidaceae*, and lower *Veillonellaceae* compared to those without NASH etiology (43). The number of *Escherichia coli*, *anaerobes*, *Lactobacillus*, and *streptococci* in intestinal microflora of patients with NAFLD is higher than that of healthy controls (44). Compared to healthy controls, the proportion of *Bacteroidetes* was significantly reduced, whereas *Proteobacteria* and *Fusobacteria* were highly enriched in the ARLC patients with different etiologies (45). The median abundance of *Bacteroidetes* was lower and the median abundance of *Proteobacteria* was higher in the intestines of patients with ALD, and these changes appeared to be associated with higher serum endotoxin levels in some of the samples (46). Studies have shown that alcohol causes a significant increase in *Veillonellaceae* and a decreasing trend in *Bacteroidaceae* and *Porphyromonadaceae* (47). 16S rRNA gene sequencing revealed that *Peptostreptococcacae*, *Proteobacteria*, *Pasteurellaceae* and *Bacillales* were significantly increased, while *Lachnospiraceae*, *Ruminococcaeae*, *Clostridiales cluster XIV*, *Prevotellaceae* and *Bacteroidaceae* significantly decreased in ARLC patients (48). Metagenomic sequencing revealed that *Bifidobacterium*, *Streptococcus* and *Lactobacillusspecies* were significantly increased, while *Akkermansia*, *Coprococcus*, *Unclassified* and *Clostridiales* significantly decreased in ARLC patients (49).

For NASH patients, there was a significant increase in *Clostridium coccoides*, Porphyromonadaceae and Actioidaceae and a decrease in Veillonellaceae and Bacteroidetes (43, 50). For NAFLD patients, *ClostridiumXI* in the Peptostreptococcaceae, the *Anaerobacter* in the Clostridiaceae, *Streptococcus* and *Lactobacillus* were significantly increased, while *Lentisphaerae*, Ruminococcaceae, *Oscillibacter*, *Flavonifractor* and Bacteroidetes decreased (44). Table 1 summarizes the studies about gut dysbiosis in ALD and NAFLD-related liver disease.

**Table 1:** The changed gut microbiota in alcohol and NASH-related liver disease.

Author year	Study population	Study method samples	LC cases	Changed microbiota	
				Increased	Decreased
Chen et al. (45) 2011	36 patients 24 HCs	16S rRNA gene sequencing Stool samples	12 ARLC	Proteobacteria, Fusobacteria *Enterobacteriaceae* *Veillonellaceae* *Streptococcaceae*	Bacteroidetes *Lachnospiraceae*
Mutlu et al. (46) 2012	48 patients 18 HCs	LH-PCR Mucosal tissue	19 ARLC	Firmicutes *Bacilliand* *Gammaprotoebacteria*	Bacteroidetes *Clostridia*
Kakiyama et al. (47) 2013	84 patients 19 HCs	16S rRNA gene sequencing Mucosal tissue	7 ARLC	*Enterobacteriaceae*	*Lachonospiraceae* *Ruminococcaceae* *Blautia*
Mouzaki et al. (50) 2013	33 patients 17 HCs	qPCR Stool samples	22 NASH	*C. coccoides*	*Bacteroidetes*
Raman et al. (51) 2013	30 patients 30 HCs	16S rRNA gene sequencing Stool samples	30 NAFLD	Firmicutes *Lachnospiraceae* *Dorea*, *Robinsoniella Roseburia* *Lactobacillus*	*Ruminococcaceae* *Porphyromonadaceae* *Oscillibacter*
Zhu et al. (52) 2013	47 patients 16 HCs	16S rRNA gene sequencing Stool samples	22 NASH	Bacteroides: *Prevotellaceae* *Prevotella* *Porphyromonadaceae porphyromonas* Proteobacteria: *Enterobacteriaceae* *Escherichia*	Firmicutes: *Lachanospiraceae* *Ruminococcaceae* *Blautiaand* *Faecalibacterium* Clostridium Actinobacteria: Bifidobacteriaceae *Bifidobacterium*
Bajaj et al. (43) 2014	219 patients 25 HCs	16S rRNA gene sequencing Stool samples	32 NASH	*Porphyromonadaceae acterioidaceae*	*Veillonellaceae*
Bajaj et al. (43) 2014	219 patients 25 HCs	16S rRNA gene sequencing Stool samples	43 ARLC	*Enterobacteriaceae* *Halomonadaceae*	*Lachnospiraceae* *Ruminococcaceae* *ClostridialiesXIV*
Jiang et al. (44) 2015	53 patients 32 HCs	16S rRNA gene sequencing Stool samples	53 NAFLD	Firmicutes: *Peptostreptococcaceae*: *ClostridiumXI* *Clostridiaceae*: *Anaerobacter* *Streptococcus* *Lactobacillus*	Lentisphaerae *Ruminococcaceae* *Oscillibacter* *Flavonifractor* Bacteroidetes: *Porphyromonadaceae* *Odoribacter* *Porphyromonadaceae* *Rikenellaceae* *Alistipes*
Bajaj et al. (48) 2017	48 patients 18 HCs	16S rRNA gene sequencing Mucosal tissue	20 ARLC	*Peptostreptococcacae* *Proteobacteria* *Pasteurellaceae* *Bacillales*	*Lachnospiraceae* *Ruminococcaeae* *Clostridiales cluster XIV* *Prevotellaceae* *Bacteroidaceae*
Dubinkina et al. (49) 2017	99 patients 60 HCs	Metagenomic Stool samples	27 ARLC	*Bifidobacterium* *Streptococcus* *Lactobacillusspecies*	*Akkermansia* *Coprococcus* *Unclassified Clostridiales*

HCs, healthy controls; 16S rRNA, 16S ribosomal RNA; LH-PCR, length heterogeneity PCR; LC, liver cirrhosis; ARLC, alcohol-related liver cirrhosis; NASH, non-alcoholic steatohepatitis; NAFLD, non-alcoholic fatty liver disease; PCR, polymerase chain reaction; LH-PCR, length heterogeneity PCR; qPCR, quantitative real-time PCR.

### 2.2 Viral hepatitis-related cirrhosis

Infection of hepatitis B and C viruses is the cause of viral hepatitis cirrhosis, which Characterized by chronic inflammation, diffuse liver fibrosis, and pseudolobular formation (53). Recent works have shown that hepatitis virus (mainly hepatitis B virus and hepatitis C virus) related cirrhosis has unique bacterial or fungal microbiota profiles, which include increased numbers of *Enterobacteriaceae*, *Prevotella*, *Streptococcus*, *Staphylococcaceae*, and *Veillonella* spp., as well as decreased *Firmicutes*, *Bifidobacteria*, *Lachnospiraceae*, *Bacteroidetes*, *Ruminococcus*, and *Clostridium* (54, 55). Sequencing of the 16S rRNA gene for HBV patients revealed significant increases in Veillonella, Megasphaera, Dialister, Atopobium, and Prevotella, and significant decreases in Neisseria, Haemophilus, and SR1 genera incertae sedis significantly decreased (56). Sequencing of the 16S rRNA gene for HCV revealed a significant increase in *Prevotella*, *Succinivibrio*, *Catenibacterium* and *Megasphaera* in the Ruminococcaceae and a significant decrease in Enterobacteriaceae, Erysipelotrichaceae and Rikenellaceae (57). But whether the direct-acting antivirals could affect the intestinal microbiota composition in cirrhotic patients is still a matter of controversy (58, 59). Although the gut microbiota varied slightly from study to study, small differences were found after treating patients. Table 2 summarizes the recent studies about gut dysbiosis in viral hepatitis-related disease.

**Table 2:** The changed gut microbiota in viral hepatitis-related liver disease.

Author year	Study population	Study methods samples	LC cases	Changed microbiota	
				Increased	Decreased
Chen et al. (45) 2011	36 patients 24 HCs	16S rRNA gene sequencing Stool samples	24 HBV	Proteobacteria Fusobacteria *Enterobacteriaceae* *Veillonellaceae* *Streptococcaceae*	Bacteroidetes *Lachnospiraceae*
Xu et al. (60) 2012	32 patients 15 HCs	PCR Stool samples	16 HBV	*Bifidobacterium* species *Bifidobacterium dentium*	*Bifidobacterium* species *Bifidobacterium longum* *Bifidobacterium* *catenulatum*
Wei et al. (61) 2013	20 patients 20 HCs	Metagenomics Stool samples	20 HBV	Proteobacteria *Enterobacteriaceae* *Veillonellaceae* *Streptococcaceae* *Escherichiacoli* *Veillonelladispar* *Veillonellaparvula*	Bacteroidetes *Bacteroides*
Qin et al. (62) 2014	123 patients 114 HCs	Metagenomics Stool samples	99 HBV	Proteobacteria Fusobacteria *Veillonella*, *Streptococcus* *Clostridium* *Prevotella*	Bacteroidetes *Bacteroides*
Bajaj et al. (43) 2014	219 patients 25 HCs	16S rRNA gene sequencing Stool samples	119 HCV	*Staphylococcae* *Enterococceae* *Enterobacteriaceae*	*Clostridiales XIV* *Lachnospiraceae* *Ruminococcaceae* *Rikenellaceae* *Veillonellaceae* *Porphyromonadaceae*
Aly et al. (63) 2016	7 patients 8 HCs	16S rRNA gene sequencing Stool samples	7 HCV	Bacteroidetes *Prevotella* *Acinetobacter* *Veillonella* *Phascolarctobacterium*	*Ruminococcus* *Parabacteroides* *Bifidobacterium*
Chen et al. (56) 2016	30 patients 20 HCs	16S rRNA gene sequencing Mucosal tissue	24 HBV	Firmicutes *Veillonella*, *Megasphaera* *Dialister* *Atopobium* *Prevotella*	Proteobacteria *Neisseria* *Haemophilus* *SR1 genera incertae sedis*
Ponziani et al. (59) 2018	12 patients 12 HCs	16S rRNA gene sequencing Stool samples	12 HCV	Proteobacteria *Staphylococcaceae* *Veillonellaceae* *Enterobacteriaceae* *Corynebacteriaceae* *Micrococcaceae* *Staphylococcus* *Dialister* *Eubacterium* *Enterococcus* *Corynebacterium*	*Methanobacteriaceae* *Methanobrevibacter*
Inoue et al. (64) 2018	166 patients 23 HCs	16S rRNA gene sequencing Stool samples	40 HCV	*Streptococcus* *Lactobacillus*	Lachnospiraceae, Ruminococcaceae
Heidrich et al. (65) 2018	95 patients 50 HCs	16S rRNA gene sequencing Stool samples	38 HCV	*Veillonella* spp. *Lactobacillus* spp. *Streptococcus* ssp. *Alloprevotella* spp. *Citrobacter* spp. *Clostridium sensustricto* spp. *Pediococcus* spp. *Akkermansia* spp. *Bifidobacterium* spp. *Escherichia/Shigella* spp. *Haemophilus* spp. *Micrococcus* spp. *Weissella* spp.	*Bilophila* spp. *Clostridium IV* spp. *Clostridium XlVb* spp. *Mitsuokella* spp. *Vampirovibrio* spp. *Butyricimonas* spp. *Victivallis* spp.
Zeng et al. (66) 2019	67 patients 15 HCs	16S rRNA gene sequencing Stool samples	25 HBV	Bacteroidetes *Bacteroides* *Akkermansia* *Atopobium* *Atopobium* *Parabacteroides*	Firmicutes *Actinobacteria*
Sultan et al. (57) 2021	38 patients 38 HCss	16S rRNA gene sequencing Stool samples	38 HCV	Ruminococcaceae *Prevotella* *Succinivibrio* *Catenibacterium* *Megasphaera*	Enterobacteriaceae Erysipelotrichaceae Rikenellaceae *Bacteroides* *Dialister* *Bilophila* *Streptococcus* *Parabacteroides* *Alistipes*

HCs, Healthy controls; 16S rRNA, 16S ribosomal RNA; LC, Liver cirrhosis; HBV, Hepatitis B Virus; HCV, Hepatitis C Virus; PCR, Polymerase Chain Reaction.

## 3 Liver cirrhosis-related complications and dysbiosis

**Figure 1:**
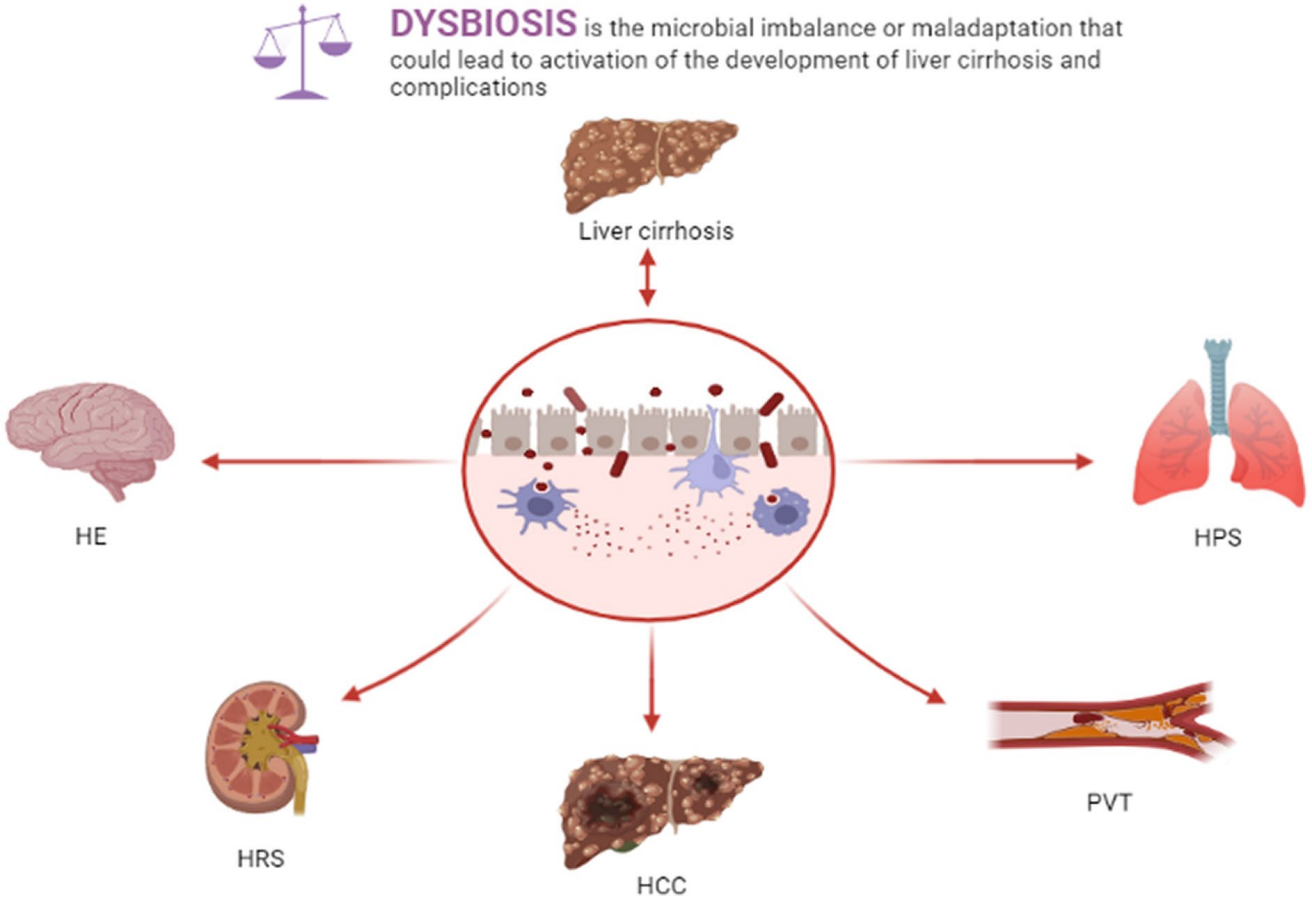
Dysbiosis and liver cirrhosis and complications.

Liver cirrhosis-related complications severely impact the survival rate and mortality of patients with liver cirrhosis, such as portal vein thrombosis (PVT), spontaneous bacterial peritonitis (SBP), hepatic encephalopathy (HE), portal hypertension (PH), hepatorenal syndrome (HRS), hepatopulmonary syndrome (HPS), and hepatocellular carcinoma (HCC). Gut dysbiosis, bacterial translocation, and intestinal barrier injury in liver cirrhosis patients exert crucial roles in liver cirrhosis-related complications (Figure 1).

### 3.1 PVT

PVT is a common complication observed in liver cirrhosis and occurs in intrahepatic branches of the portal vein, with or without superior mesenteric vein and splenic vein thrombosis (67). The prevalence of PVT is approximately 1%–26% (68). The pathogenesis of liver cirrhosis with PVT is unclear. Slow portal vein blood flow caused by liver cirrhosis is the important factor for PVT (69). The decrease of symbiotic anaerobes and the increase of pathogen abundance after gut dysbiosis, especially the increase of Gram-negative *Enterobacteriaceae*, are closely related to the occurrence of PVT (70). Bacterial LPS is the glycolipid on the outer membrane of Gram-negative bacteria and is one of the key factors in the hypercoagulable state of liver cirrhosis (71, 72). The translocation of bacteria and pathogen-associated molecular patterns (PAMPs), especially LPS, will cause systemic inflammation, endotoxemia, and platelet activation, at the same time, systemic inflammation and elevated levels of systemic inflammatory factors exacerbate the risk of PVT formation (73, 74). Liver cirrhosis patients usually have higher endotoxemia and systemic inflammation due to gut dysbiosis. Endotoxin can increase thrombosis through the production of tissue factor (TF) (75). With the increase of intestinal permeability and bacterial translocation, the level of LPS in the blood of patients with liver cirrhosis increased significantly. Due to the immune function of the liver, the level of LPS in the portal system was significantly higher than that in the systemic circulation (76, 77).

**Figure 2:**
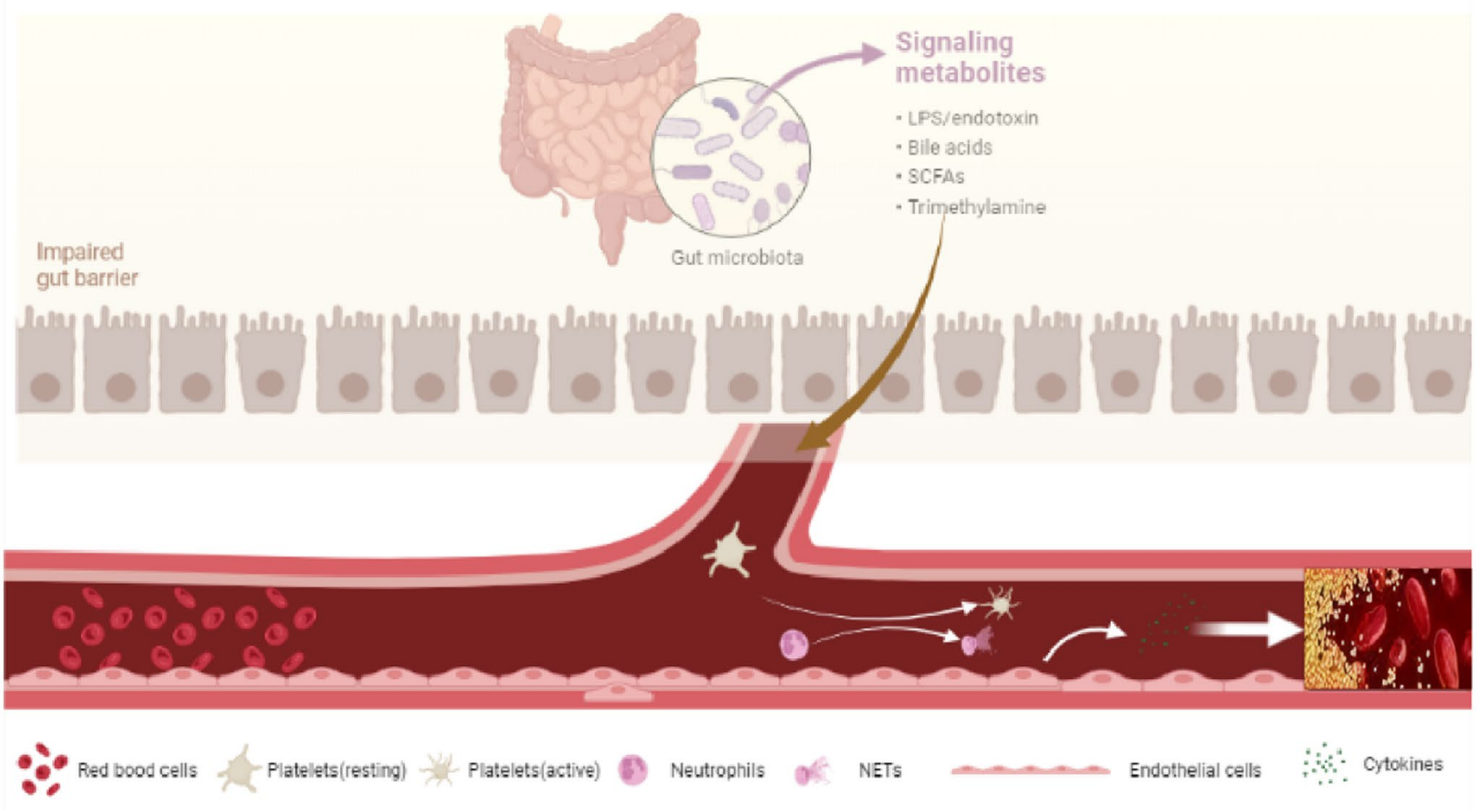
Dysbiosis and PVT.

After entering the circulation system, LPS interacts to toll-like receptors (TLRs) and initiating a series of pathophysiological changes linked to the formation of PVT. On the one hand, LPS binds to TLRs expressed on hepatocytes and immune cells. Activation of these cells will release a huge amount of inflammatory cytokines, chemokines, vasoactive factors, adhesion molecules, and reactive oxygen species (ROS) (78–80), resulting in systemic inflammation, the proliferation of hepatic stellate cells, and the development of liver cirrhosis (81). On the other hand, LPS binds to TLRs receptors expressed on vascular endothelial cells, platelet, and neutrophils, promoting blood hypercoagulability and PVT in the context of liver cirrhosis (70) (Figure 2).

#### 3.1.1 LPS and endothelial cells

LPS binds to TLRs receptors on vascular endothelial cells and activates endothelial cells to release von Willebrand factor (vWF) and factor VII (43, 82). Animal experiments showed that LPS could also bind to toll-like receptor 2 (TLR2) on hepatic endothelial cells to promote the synthesis of the vWF precursor and promote platelet integrin-dependent thrombus growth, while the synthesis of vWF in hepatic endothelial cells decreased and the level of plasma vWF decreased significantly in TLR2 knockout mice (83). *In vitro* studies have shown that LPS can stimulate the formation and secretion of Weibel–Palade bodies in endothelial cells through toll-like receptor 4 (TLR4), and promote the release of VIII and vWf into the blood resulting in a blood hypercoagulable state. TLR receptor blockers can significantly inhibit the release of VIII and vWf, indicating that LPS in the intestinal microbiota increases the level of systemic factor VIII by stimulating the release of endothelial cells (43). The level of Plasminogen activator inhibitor-1 (PAI-1) and tissue plasminogen activator (TPA), key determinants in thrombosis, were shown to be related to the gut microbiota. *In vivo* and *in vitro* studies have shown that bacterial lipoprotein TLR2 agonists can increase vascular endothelial cell permeability, increase plasma PAI-1 and decrease TPA (84).

#### 3.1.2 LPS and platelets

Platelet activation in patients with liver cirrhosis may be associated with increased levels of circulating LPS (85). TLR2 and TLR4 are widely distributed on the surface of platelets. The activation of the TLR2 in platelets causes the activation of platelets, which can cause platelets to release their own stores of α-particles and dense particles to interact with vascular endothelial cells and promote platelet-dependent thrombosis (86, 87). *In vitro* studies have shown that platelets in patients with liver cirrhosis are more reactive to TLRs agonists than healthy people and have nothing to do with the number of platelets. The reactivity is significantly weakened in the presence of TLR4 blockers, indicating that LPS may activate platelets through TLR4 and may lead to liver cirrhosis-related thrombotic complications (70). Activated platelets release P-selectin to stimulate monocytes and macrophages to release chemokines, promote platelet-monocyte aggregation, and release inflammatory molecules to change the chemotaxis and adhesion of endothelial cells (88).

#### 3.1.3 LPS and neutrophils

Activation of neutrophils can induce thrombosis through the neutrophil extracellular bactericidal networks (NETs) (89, 90). *In vitro* animal models have shown that the elimination of NETs can prevent thrombosis (91). NETs are formed by neutrophils releasing nuclear contents (DNA-histone complex, double-stranded DNA, and neutrophil elastase) into extracellular space (92). Histone-DNA complex can activate coagulation factor XIIa activated by coagulation factor XII, and then activate coagulation factor XI and downstream endogenous coagulation pathway (93). Histone of NETs can promote thrombin production, endothelial cell activation, and thrombus formation through the platelet-dependent mechanism mediated by TLR2 and TLR4 (94).

#### 3.1.4 Trimethylamine-N-oxide

Trimethylamine (TMA) lyase from intestinal microorganisms metabolizes phosphatidylcholine, choline and carnitine to TMA, which is further processed to trimethylamine-N-oxide (TMAO) by flavin monooxygenase (FMO) in the liver (95). TMAO not only aggravates cardiovascular events but also is closely related to the formation of thrombosis (96). TMAO activates platelets by increasing the release of Ca^2+^ stored platelets intracellular, while platelets with hyperreactivity enhance the thrombosis risk (97). The animal experiment showed that a high choline diet can enhance platelet hyperresponsiveness, but this will not occur with the intervention of antibiotics or a high choline diet in germ-free mice (98). Therefore, TMAO acts as a medium to closely connect intestinal microbiota with thrombosis.

Liver cirrhosis is usually accompanying by acquired factors of thrombophilia, such as hyperhomocysteinemia, secondary to vitamin B and folate deficiencies, and antiphospholipid antibody syndrome (67). Moreover, the levels of albumin and protein C were lower in patients with liver cirrhosis compared to healthy controls, and low levels of albumin and protein C were associated with the increased risk of PVT formation (99, 100). Abnormal metabolic state, hemodynamic changes, and PAI-1 in patients with NAFLD may contribute to prethrombotic state and hypercoagulable state (101, 102). In a cross-sectional study, NASH-related cirrhosis was the strongest independent risk factor for the independent diagnosis of PVT in patients undergoing liver transplantation (103).

### 3.2 SBP

SBP is a common and serious complication in cirrhotic patients (104). It refers to primary peritonitis that occurs in patients with cirrhosis and ascites without abdominal infection lesions (105). Animal experiments showed that the bacteria strain isolated from intestinal mucosa lymphoid tissue and ascites was similar (106). These studies provide strong evidence for the role of bacterial translocation in SBP. The bacterial translocation and the increase of intestinal permeability is the main mechanism of SBP in the setting of liver cirrhosis, at the same time, the decrease of host immune clearance ability is also closely related to the occurrence of SBP (43).

Gram-negative bacteria, such as *Escherichia coli* and *Klebsiella*, and Gram-positive bacteria are common causes of SBP (107, 108). The patients with liver cirrhosis were accompanied by decreased diversity of the gut microbiota, which was characterized by a significant reduction in autochthonous taxa and a significant increase in pathogenic taxa (109). Clinical studies have shown that *Streptococcus* spp., *Klebsiella*, *Escherichia*, and *Citrobacter* spp. were mainly infectious organisms on routine culture in liver cirrhosis patients with SBP (43).

The gut-liver-immune axis plays a key role in SBP. The changes in intestinal motility, mucosal immunity, and drug usage in patients with liver cirrhosis will lead to significant changes in the composition of gastrointestinal microorganisms and aggravate the translocation of the intestinal microbiota (110, 111). Moreover, interaction between BA and its receptor farnesoid X receptor (FXR) helps to maintain intestinal barrier function and reduce bacterial translocation (112). Animal experiments showed that FXR agonists could reduce intestinal permeability and bacterial translocation through FXR (29, 113). In addition to FXR agonists, selective intestinal decontamination is an important measure for the treatment of SBP. Intestinal decontamination with rifaximin and norfloxacin significantly decreased the incidence of SBP in cirrhotic patients with ascites (114, 115), but rifaximin was more effective than norfloxacin in the secondary prevention of SBP (116). Moreover, non-selective β-blockers (NSBB) can reduce intestinal permeability, markers of bacterial translocation (IL-6/LPS binding protein), and SBP by improving intestinal motility and reducing intestinal bacterial overgrowth (117, 118).

### 3.3 HE

HE is a severe complication of advanced liver cirrhosis and is closely linked to the gut-liver-brain axis (119). The impaired hepatic clearance ability with the progression of liver cirrhosis is reconsider phrasing for clarity, neurotoxic substances, and false neurotransmitters produced by the intestinal microbiota (120). These substances have an important effect on HE. Moreover, the formation of portal shunts further facilitates the entrance of the microbial metabolites into the blood (121).

A series of inflammatory cytokines and endotoxemia caused by gut dysbiosis can impair blood-brain barriers, neuroinflammation, and affect cognition (122). Compared with healthy people, the specific bacteria (*Alcaligenes*, *Porphyromonas*, and *Enterobacteriaceae*) in fecal microbiota were significantly increased in liver cirrhosis patients complicated with HE and were strongly related to the cognition and inflammation of HE (123). A study analyzed the relationship between cognition, Magnetic resonance imaging parameters, and intestinal microflora and found that patients with HE had a significantly lower cognitive ability, systemic inflammation, gut dysbiosis, and hyperammonemia than controls and cirrhotic patients without HE (119). Specific microbial families (autochthonous taxa negatively and Enterobacteriaceae positively) correlated with changes in astrocytes associated with magnetic resonance spectroscopy and hyperammonemia (119). Treatment methods such as fecal microbial transplantation, intestinal decontamination, and diet regulation for the intestinal microbiota can improve the cognitive level of patients with HE (124–126). Therefore, Gut dysbiosis is associated with the development of HE (43).

Emerging evidence also shows that brain cholesterol accumulation contributes to the progression of HE through BA-mediated effects on the FXR (127). Moreover, serotonin and tryptophan metabolism via the gut microbiota is a key factor for the occurrence of central nervous system diseases (128). Recent studies have found that bacteria (*Stenotrophomonas pavanii*, *Methylobacterium extorquens*) and metabolites (methanol, threonine) in the blood and feces of patients with liver cirrhosis are positively correlated with HE, while fecal *Enterobacteriaceae* and TMA were positively correlated with blood proinflammatory cytokines (129). Therefore, bacteria or their metabolites in the blood are correlated with systemic inflammation and HE in patients with liver cirrhosis. Those studies provide new perspectives and treatment strategies for the pathogenesis of HE.

### 3.4 PH

Liver structural disorder and nodular regeneration in liver cirrhosis will cause compression of hepatic sinusoids and blood vessels, and increase intrahepatic resistance, resulting in (PH) (130). PH has an important effect on intestinal permeability, gut microbiota, and bacterial translocation. PH reduces intestinal mucosal blood flow, causes neoangiogenesis, ischemia, and edema of the intestine, destroying intestinal barrier function (131). Therefore, the gut dysbiosis in liver cirrhosis causes the translocation of a large number of bacteria and products, especially LPS, into the blood, leading to activate the immune system and causes systemic inflammation via interacting with TLRs (43, 132), and activation of the liver immune system and systemic inflammation promote the progression of liver cirrhosis and aggravate PH (133). In the animal model, intraperitoneal injection of LPS activates the expression of TLR4 and increases inflammatory mediators, leading to escalating PH (134). The release of systemic inflammatory factors (nitric oxide, NO), can also reduce systemic vascular resistance and induce hyperdynamic circulation, thus affecting PH (135, 136). Therefore, there is an important pathophysiological relationship between PH and the gut-liver axis.

FXR not only plays an important role in intestinal microflora homeostasis, BA metabolism, and intestinal barrier function, but also in anti-fibrosis and reducing PH. In the experimental liver cirrhosis, non-steroidal FXR agonist PX20606 improves PH via reducing intestinal bacterial migration, liver fibrosis, vascular remodeling, and hepatic sinusoid dysfunction (137). Studies in preclinical models of cirrhosis shows that intestinal decontamination can improve the portal vein pressure and hyperdynamic circulation in liver cirrhosis (138). These studies provide insight into molecular mechanisms and novel therapeutic targets in PH.

### 3.5 HRS

HRS refers to functional acute renal failure in patients with severe liver disease (139). HRS is a severe complication of advanced liver cirrhosis with a prevalence between 11% and 20% (140). The mechanism of HRS is not clear, but it is mainly related to the following two aspects: on the one hand, the production of ascites increases, and the circulating blood volume decreases after decompensation of liver cirrhosis, resulting in prerenal renal failure (141); on the other hand, the bacterial dysbiosis and the translocation of bacteria and related products after decompensation of liver cirrhosis lead to the endotoxemia, which is closely related to HRS (142, 143). Animal model studies have shown that the increased expression of TLR4 in the kidney tissue of cirrhotic rats increases the susceptibility to LPS, then activates the NF-κB pathway, increases the expression of proinflammatory cytokine tumor necrosis factor-α (TNF-α), and renal tubular injury (144). Selective gut decontamination can improve the systemic hemodynamics and renal function of patients with liver cirrhosis, indicating that the gut microbiota plays an important role in HRS (144, 145). Moreover, the albumin infusion improves renal function in cirrhotic patients and sepsis via affecting endotoxemia, hemodynamics, and oxidative stress (146, 147). Moreover, circadian hemodynamic in cirrhosis is related to renal function (148). As mentioned above, gut dysbiosis has a close relationship with circadian rhythm, it is not clear whether the diurnal rhythm of the microbiota can affect renal function by regulating hemodynamics.

### 3.6 HPS

HPS is a pulmonary complication in liver cirrhosis patients and charactered by pulmonary microvascular dilatation and hypoxemia (149). The pathogenesis of HPS is unknown. The bacterial translocation, intestinal endotoxemia, and pulmonary microvascular dilatation may closely relate to the pathogenesis of HPS (150–152). Studies on animal models have shown that bacterial translocation, intestinal endotoxemia, and related inflammatory factors are closely related to the occurrence of HPS (153). Moreover, bacterial translocation can increase the incidence and severity of HPS in cirrhotic rats, and prophylactic norfloxacin usage can reduce the incidence and severity of HPS (154). However, antibiotic norfloxacin usage in clinical patients does not improve the gas exchange of HPS (155). Therefore, there need to be further studies on the effect of antibiotics in patients with HPS and the role of the specific intestinal microbiota in the pathogenesis of HPS.

### 3.7 HCC

HCC is the most common type of liver cancer as well as the common cause of death in patients with advanced cirrhosis (156, 157). Moreover, different etiologies may affect the composition of the microbiota in HCC patients (158). As aforementioned, systemic inflammation caused by the translocation of bacteria, LPS, bacterial DNA, and peptidoglycans by activating the TLRs is crucial for the development of HCC (42, 159). LPS activates TLR4 signal to promote the production of interleukin-6 (IL-6) and TNF-α, mediating the differentiation of hepatic progenitor cells into myofibroblasts, and promoting the proliferation and malignant transformation of hepatic progenitor cells (160). Therefore, LPS and TLR4 are closely related to the development of cirrhotic patients to HCC (161). Compared to NAFLD cirrhotic patients without HCC, the NAFLD cirrhotic patients with HCC have a higher abundance of *Bacteroidetes* at the phylum level, *Bacteroidaceae*, *Streptococcaceae*, *Enterococcaceae*, and *Gemellaceae* at the family level, and *Phascolarctobacterium*, *Enterococcus*, *Streptococcus*, *Gemella*, and *Bilophila* at the genus level (162). In another study, intestinal microbiota constitution in cirrhotic patients with HCC is distinguished from those without HCC. Compared patients without HCC, *Haemophilus*, *Eggerthella*, *Bifidobacterium*, *Butyricimonas*, *Christensella*, *Odoribacter*, an unknown genus phylum *Tenericutes*, and an unknown genus, phylum *Firmicutes*, family *Erysipelotrichaceae* were all elevated in cirrhotic patients with HCC, while *Fusobacterium*, *Prevotella*, *Streptococcus*, *S24-7* (Phylum *Bacteroidetes*) and an unknown genus were all decreased. Thus, gut dysbiosis is a crucial factor in cirrhotic patients with HCC (163).

## 4 Conclusion

The intestinal microbiota plays a significant role in human health and disease. Gut dysbiosis is associated with the onset and progression of liver cirrhotic and its complications. The correlation between specific intestinal microbiota and pathogenesis of liver cirrhosis related complications needs further study. The gut microbiota can be used as a potential diagnosis biomarker and treatment target for liver cirrhosis and its complications. It has been reported that bacteriophage therapy (164), microRNA therapy (165), and carbon nanoparticles (166) that based on targeting the intestinal microbiota in liver cirrhosis. It is important to assess the role of the gut microbiota in the pathogenesis of liver cirrhosis and its complications. With the rise of personalized medicine and nanomedical technology, treatment options that targeting specific intestinal microbiota composition may be the most promising treatment for liver cirrhosis and complications in the future.

## Author contributions

GN: Conceptualization. HZ: Conceptualization, Data curation, Writing – original draft. DX: Conceptualization, Data curation, Writing – original draft. JY: Investigation, Writing – review & editing. XL: Project administration, Supervision, Writing – review & editing.

## References

[1] Fleming KM Aithal GP Card TR West J. All-cause mortality in people with cirrhosis compared with the general population: a population-based cohort study. Liver Int. (2012) 32:79–84. doi: 10.1111/j.1478-3231.2011.02517.x, PMID: 21745279

[2] Ginès P Krag A Abraldes JG Solà E Fabrellas N Kamath PS. Liver cirrhosis. Lancet. (2021) 398:1359–76. doi: 10.1016/S0140-6736(21)01374-X 34543610

[3] Pimpin L Cortez-Pinto H Negro F Corbould E Lazarus JV Webber L. Burden of liver disease in Europe: epidemiology and analysis of risk factors to identify prevention policies. J Hepatol. (2018) 69:718–35. doi: 10.1016/j.jhep.2018.05.011, PMID: 29777749

[4] Xiao J Wang F Wong NK He J Zhang R Sun R. Global liver disease burdens and research trends: analysis from a Chinese perspective. J Hepatol. (2019) 71:212–21. doi: 10.1016/j.jhep.2019.03.004, PMID: 30871980

[5] Parola M Pinzani M. Liver fibrosis: pathophysiology, pathogenetic targets and clinical issues. Mol Aspects Med. (2019) 65:37–55. doi: 10.1016/j.mam.2018.09.002 30213667

[6] Albillos A de Gottardi A Rescigno M. The gut-liver axis in liver disease: pathophysiological basis for therapy. J Hepatol. (2020) 72:558–77. doi: 10.1016/j.jhep.2019.10.003, PMID: 31622696

[7] Sender R Fuchs S Milo R. Revised estimates for the number of human and bacteria cells in the body. PLoS Biol. (2016) 14:e1002533. doi: 10.1371/journal.pbio.1002533, PMID: 27541692 PMC4991899

[8] Blum HE. The human microbiome. Adv Med Sci. (2017) 62:414–20. doi: 10.1016/j.advms.2017.04.005 28711782

[9] Kennedy KM Gerlach MJ Adam T Heimesaat MM Rossi L Surette MG. Fetal meconium does not have a detectable microbiota before birth. Nat Microbiol. (2021) 6:865–73. doi: 10.1038/s41564-021-00904-0 33972766

[10] Schoeler M Caesar R. Dietary lipids, gut microbiota and lipid metabolism. Rev Endocr Metab Disord. (2019) 20:461–72. doi: 10.1007/s11154-019-09512-0, PMID: 31707624 PMC6938793

[11] Dinsmoor AM Aguilar-Lopez M Khan NA Donovan SM. A systematic review of dietary influences on fecal microbiota composition and function among healthy humans 1–20 years of age. Adv Nutr. (2021) 12:1734–50. doi: 10.1093/advances/nmab047, PMID: 33951139 PMC8483965

[12] Han Q Wang J Li W Chen ZJ Du Y. Androgen-induced gut dysbiosis disrupts glucolipid metabolism and endocrinal functions in polycystic ovary syndrome. Microbiome. (2021) 9:101. doi: 10.1186/s40168-021-01046-5, PMID: 33957990 PMC8103748

[13] Wang Z Chen WH Li SX He ZM Zhu WL Ji YB. Gut microbiota modulates the inflammatory response and cognitive impairment induced by sleep deprivation. Mol Psychiatry. (2021) 26:6277–92. doi: 10.1038/s41380-021-01113-1 33963281

[14] Li J Jia H Cai X Zhong H Feng Q Sunagawa S. An integrated catalog of reference genes in the human gut microbiome. Nat Biotechnol. (2014) 32:834–41. doi: 10.1038/nbt.2942, PMID: 24997786

[15] Zhou R Fan X Schnabl B. Role of the intestinal microbiome in liver fibrosis development and new treatment strategies. Transl Res. (2019) 209:22–38. doi: 10.1016/j.trsl.2019.02.005, PMID: 30853445

[16] Mazagova M Wang L Anfora AT Wissmueller M Lesley SA Miyamoto Y. Commensal microbiota is hepatoprotective and prevents liver fibrosis in mice. FASEB J. (2015) 29:1043–55. doi: 10.1096/fj.14-259515, PMID: 25466902 PMC4422368

[17] Hamoud AR Weaver L Stec DE Hinds TD Jr. Bilirubin in the liver-gut signaling axis. Trends Endocrinol Metab. (2018) 29:140–50. doi: 10.1016/j.tem.2018.01.002, PMID: 29409713 PMC5831340

[18] Inagaki T Moschetta A Lee YK Peng L Zhao G Downes M. Regulation of antibacterial defense in the small intestine by the nuclear bile acid receptor. Proc Natl Acad Sci U S A. (2006) 103:3920–5. doi: 10.1073/pnas.0509592103, PMID: 16473946 PMC1450165

[19] Long SL Gahan C Joyce SA. Interactions between gut bacteria and bile in health and disease. Mol Aspects Med. (2017) 56:54–65. doi: 10.1016/j.mam.2017.06.002, PMID: 28602676

[20] Munteanu D Negru A Radulescu M Mihailescu R Arama SS Arama V. Evaluation of bacterial translocation in patients with chronic HCV infection. Rom J Intern Med. (2014) 52:91–6. PMID: 25338345

[21] Acharya C Sahingur SE Bajaj JS. Microbiota, cirrhosis, and the emerging oral-gut-liver axis. JCI Insight. (2017) 2:e94416. doi: 10.1172/jci.insight.94416 28978799 PMC5841881

[22] Wahlström A Sayin SI Marschall HU Bäckhed F. Intestinal crosstalk between bile acids and microbiota and its impact on host metabolism. Cell Metab. (2016) 24:41–50. doi: 10.1016/j.cmet.2016.05.005, PMID: 27320064

[23] Di Ciaula A Garruti G Lunardi Baccetto R Molina-Molina E Bonfrate L Wang DQ. Bile acid physiology. Ann Hepatol. (2017) 16:S4–S14. doi: 10.5604/01.3001.0010.5493 29080336

[24] Macierzanka A Torcello-Gómez A Jungnickel C Maldonado-Valderrama J. Bile salts in digestion and transport of lipids. Adv Colloid Interf Sci. (2019) 274:102045. doi: 10.1016/j.cis.2019.102045 31689682

[25] Bauer TM Schwacha H Steinbrückner B Brinkmann FE Ditzen AK Aponte JJ. Small intestinal bacterial overgrowth in human cirrhosis is associated with systemic endotoxemia. Am J Gastroenterol. (2002) 97:2364–70. doi: 10.1111/j.1572-0241.2002.05791.x, PMID: 12358257

[26] Swann JR Want EJ Geier FM Spagou K Wilson ID Sidaway JE. Systemic gut microbial modulation of bile acid metabolism in host tissue compartments. Proc Natl Acad Sci U S A. (2011) 108:4523–30. doi: 10.1073/pnas.1006734107, PMID: 20837534 PMC3063584

[27] Li T Chiang JY. Bile acid signaling in metabolic disease and drug therapy. Pharmacol Rev. (2014) 66:948–83. doi: 10.1124/pr.113.008201, PMID: 25073467 PMC4180336

[28] Verbeke L Farre R Verbinnen B Covens K Vanuytsel T Verhaegen J. The FXR agonist obeticholic acid prevents gut barrier dysfunction and bacterial translocation in cholestatic rats. Am J Pathol. (2015) 185:409–19. doi: 10.1016/j.ajpath.2014.10.009, PMID: 25592258

[29] Úbeda M Lario M Muñoz L Borrero MJ Rodríguez-Serrano M Sánchez-Díaz AM. Obeticholic acid reduces bacterial translocation and inhibits intestinal inflammation in cirrhotic rats. J Hepatol. (2016) 64:1049–57. doi: 10.1016/j.jhep.2015.12.010, PMID: 26723896

[30] Usami M Miyoshi M Yamashita H. Gut microbiota and host metabolism in liver cirrhosis. World J Gastroenterol. (2015) 21:11597–608. doi: 10.3748/wjg.v21.i41.11597 26556989 PMC4631963

[31] Milosevic I Vujovic A Barac A Djelic M Korac M Radovanovic Spurnic A. Gut-liver axis, gut microbiota, and its modulation in the management of liver diseases: a review of the literature. Int J Mol Sci. (2019) 20:395. doi: 10.3390/ijms20020395, PMID: 30658519 PMC6358912

[32] Fang J Yu CH Li XJ Yao JM Fang ZY Yoon SH. Gut dysbiosis in nonalcoholic fatty liver disease: pathogenesis, diagnosis, and therapeutic implications. Front Cell Infect Microbiol. (2022) 12:997018. doi: 10.3389/fcimb.2022.997018, PMID: 36425787 PMC9679376

[33] Bajaj JS Heuman DM Hylemon PB Sanyal AJ White MB Monteith P. Altered profile of human gut microbiome is associated with cirrhosis and its complications. J Hepatol. (2014) 60:940–7. doi: 10.1016/j.jhep.2013.12.019, PMID: 24374295 PMC3995845

[34] Xu R Tan C He Y Wu Q Wang H Yin J. Dysbiosis of gut microbiota and short-chain fatty acids in encephalitis: a Chinese pilot study. Front Immunol. (2020) 11:1994. doi: 10.3389/fimmu.2020.01994, PMID: 32973805 PMC7468513

[35] Vallianou N Christodoulatos GS Karampela I Tsilingiris D Magkos F Stratigou T. Understanding the role of the gut microbiome and microbial metabolites in non-alcoholic fatty liver disease: current evidence and perspectives. Biomol Ther. (2021) 12:56. doi: 10.3390/biom12010056, PMID: 35053205 PMC8774162

[36] Brunt EM Wong VW Nobili V Day CP Sookoian S Maher JJ. Nonalcoholic fatty liver disease. Nat Rev Dis Primers. (2015) 1:15080. doi: 10.1038/nrdp.2015.80 27188459

[37] Keshavarzian A Farhadi A Forsyth CB Rangan J Jakate S Shaikh M. Evidence that chronic alcohol exposure promotes intestinal oxidative stress, intestinal hyperpermeability and endotoxemia prior to development of alcoholic steatohepatitis in rats. J Hepatol. (2009) 50:538–47. doi: 10.1016/j.jhep.2008.10.028, PMID: 19155080 PMC2680133

[38] Dunagan M Chaudhry K Samak G Rao RK. Acetaldehyde disrupts tight junctions in Caco-2 cell monolayers by a protein phosphatase 2A-dependent mechanism. Am J Physiol Gastrointest Liver Physiol. (2012) 303:G1356–64. doi: 10.1152/ajpgi.00526.2011, PMID: 23064762 PMC4073985

[39] Boursier J Mueller O Barret M Machado M Fizanne L Araujo-Perez F. The severity of nonalcoholic fatty liver disease is associated with gut dysbiosis and shift in the metabolic function of the gut microbiota. Hepatology. (2016) 63:764–75. doi: 10.1002/hep.28356, PMID: 26600078 PMC4975935

[40] Safari Z Gérard P. The links between the gut microbiome and non-alcoholic fatty liver disease (NAFLD). Cell Mol Life Sci. (2019) 76:1541–58. doi: 10.1007/s00018-019-03011-w 30683985 PMC11105223

[41] Quigley EM Stanton C Murphy EF. The gut microbiota and the liver. Pathophysiological and clinical implications. J Hepatol. (2013) 58:1020–7. doi: 10.1016/j.jhep.2012.11.023, PMID: 23183530

[42] Tsiaoussis GI Assimakopoulos SF Tsamandas AC Triantos CK Thomopoulos KC. Intestinal barrier dysfunction in cirrhosis: current concepts in pathophysiology and clinical implications. World J Hepatol. (2015) 7:2058–68. doi: 10.4254/wjh.v7.i17.2058, PMID: 26301048 PMC4539399

[43] Carnevale R Raparelli V Nocella C Bartimoccia S Novo M Severino A. Gut-derived endotoxin stimulates factor VIII secretion from endothelial cells. Implications for hypercoagulability in cirrhosis. J Hepatol. (2017) 67:950–6. doi: 10.1016/j.jhep.2017.07.002, PMID: 28716745

[44] Jiang W Wu N Wang X Chi Y Zhang Y Qiu X. Dysbiosis gut microbiota associated with inflammation and impaired mucosal immune function in intestine of humans with non-alcoholic fatty liver disease. Sci Rep. (2015) 5:8096. doi: 10.1038/srep08096 25644696 PMC4314632

[45] Chen Y Yang F Lu H Wang B Chen Y Lei D. Characterization of fecal microbial communities in patients with liver cirrhosis. Hepatology. (2011) 54:562–72. doi: 10.1002/hep.24423 21574172

[46] Mutlu EA Gillevet PM Rangwala H Sikaroodi M Naqvi A Engen PA. Colonic microbiome is altered in alcoholism. Am J Physiol Gastrointest Liver Physiol. (2012) 302:G966–78. doi: 10.1152/ajpgi.00380.2011, PMID: 22241860 PMC3362077

[47] Kakiyama G Hylemon PB Zhou H Pandak WM Heuman DM Kang DJ. Colonic inflammation and secondary bile acids in alcoholic cirrhosis. Am J Physiol Gastrointest Liver Physiol. (2014) 306:G929–37. doi: 10.1152/ajpgi.00315.2013, PMID: 24699327 PMC4152166

[48] Bajaj JS Kakiyama G Zhao D Takei H Fagan A Hylemon P. Continued alcohol misuse in human cirrhosis is associated with an impaired gut-liver axis. Alcohol Clin Exp Res. (2017) 41:1857–65. doi: 10.1111/acer.13498 28925102

[49] Dubinkina VB Tyakht AV Odintsova VY Yarygin KS Kovarsky BA Pavlenko AV. Links of gut microbiota composition with alcohol dependence syndrome and alcoholic liver disease. Microbiome. (2017) 5:141. doi: 10.1186/s40168-017-0359-2, PMID: 29041989 PMC5645934

[50] Mouzaki M Comelli EM Arendt BM Bonengel J Fung SK Fischer SE. Intestinal microbiota in patients with nonalcoholic fatty liver disease. Hepatology. (2013) 58:120–7. doi: 10.1002/hep.26319 23401313

[51] Raman M Ahmed I Gillevet PM Probert CS Ratcliffe NM Smith S. Fecal microbiome and volatile organic compound metabolome in obese humans with nonalcoholic fatty liver disease. Clin Gastroenterol Hepatol. (2013) 11:868–875.e3. doi: 10.1016/j.cgh.2013.02.015, PMID: 23454028

[52] Zhu L Baker SS Gill C Liu W Alkhouri R Baker RD. Characterization of gut microbiomes in nonalcoholic steatohepatitis (NASH) patients: a connection between endogenous alcohol and NASH. Hepatology. (2013) 57:601–9. doi: 10.1002/hep.26093, PMID: 23055155

[53] Tsochatzis EA Bosch J Burroughs AK. Liver cirrhosis. Lancet. (2014) 383:1749–61. doi: 10.1016/S0140-6736(14)60121-5 24480518

[54] Preveden T Scarpellini E Milić N Luzza F Abenavoli L. Gut microbiota changes and chronic hepatitis C virus infection. Expert Rev Gastroenterol Hepatol. (2017) 11:813–9. doi: 10.1080/17474124.2017.1343663, PMID: 28621554

[55] Wang Y Pan CQ Xing H. Advances in gut microbiota of viral hepatitis cirrhosis. Biomed Res Int. (2019) 2019:1–9. doi: 10.1155/2019/9726786 PMC6893240 31886272

[56] Chen Y Ji F Guo J Shi D Fang D Li L. Dysbiosis of small intestinal microbiota in liver cirrhosis and its association with etiology. Sci Rep. (2016) 6:34055. doi: 10.1038/srep34055, PMID: 27687977 PMC5043180

[57] Sultan S El-Mowafy M Elgaml A El-Mesery M El Shabrawi A Elegezy M. Alterations of the treatment-naive gut microbiome in newly diagnosed hepatitis C virus infection. ACS Infect Dis. (2021) 7:1059–68. doi: 10.1021/acsinfecdis.0c00432, PMID: 33119247

[58] Bajaj JS Sterling RK Betrapally NS Nixon DE Fuchs M Daita K. HCV eradication does not impact gut dysbiosis or systemic inflammation in cirrhotic patients. Aliment Pharmacol Ther. (2016) 44:638–43. doi: 10.1111/apt.13732 27417456

[59] Ponziani FR Putignani L Paroni Sterbini F Petito V Picca A Del Chierico F. Influence of hepatitis C virus eradication with direct-acting antivirals on the gut microbiota in patients with cirrhosis. Aliment Pharmacol Ther. (2018) 48:1301–11. doi: 10.1111/apt.15004, PMID: 30345704

[60] Xu M Wang B Fu Y Chen Y Yang F Lu H. Changes of fecal Bifidobacterium species in adult patients with hepatitis B virus-induced chronic liver disease. Microb Ecol. (2012) 63:304–13. doi: 10.1007/s00248-011-9925-5, PMID: 21814872

[61] Wei X Yan X Zou D Yang Z Wang X Liu W. Abnormal fecal microbiota community and functions in patients with hepatitis B liver cirrhosis as revealed by a metagenomic approach. BMC Gastroenterol. (2013) 13:175. doi: 10.1186/1471-230X-13-175, PMID: 24369878 PMC3878425

[62] Qin N Yang F Li A Prifti E Chen Y Shao L. Alterations of the human gut microbiome in liver cirrhosis. Nature. (2014) 513:59–64. doi: 10.1038/nature13568 25079328

[63] Aly AM Adel A El-Gendy AO Essam TM Aziz RK. Gut microbiome alterations in patients with stage 4 hepatitis C. Gut Pathog. (2016) 8:42. doi: 10.1186/s13099-016-0124-2, PMID: 27625705 PMC5020480

[64] Inoue T Nakayama J Moriya K Kawaratani H Momoda R Ito K. Gut dysbiosis associated with hepatitis C virus infection. Clin Infect Dis. (2018) 67:869–77. doi: 10.1093/cid/ciy205 29718124

[65] Heidrich B Vital M Plumeier I Döscher N Kahl S Kirschner J. Intestinal microbiota in patients with chronic hepatitis C with and without cirrhosis compared with healthy controls. Liver Int. (2018) 38:50–8. doi: 10.1111/liv.13485, PMID: 28561276

[66] Zeng Y Chen S Fu Y Wu W Chen T Chen J. Gut microbiota dysbiosis in patients with hepatitis B virus-induced chronic liver disease covering chronic hepatitis, liver cirrhosis and hepatocellular carcinoma. J Viral Hepat. (2020) 27:143–55. doi: 10.1111/jvh.13216, PMID: 31600845

[67] Hepatobiliary Disease Study Group, Chinese Society of Gastroenterology, Association CM. Consensus for management of portal vein thrombosis in liver cirrhosis. J Dig Dis. (2020) 22:176–86. doi: 10.1111/1751-2980.12970, PMID: 33470535 PMC8252415

[68] Loudin M Ahn J. Portal vein thrombosis in cirrhosis. J Clin Gastroenterol. (2017) 51:579–85. doi: 10.1097/MCG.0000000000000834 28489645

[69] Stine JG Wang J Shah PM Argo CK Intagliata N Uflacker A. Decreased portal vein velocity is predictive of the development of portal vein thrombosis: a matched case-control study. Liver Int. (2018) 38:94–101. doi: 10.1111/liv.13500, PMID: 28632958 PMC10540634

[70] Hasan RA Koh AY Zia A. The gut microbiome and thromboembolism. Thromb Res. (2020) 189:77–87. doi: 10.1016/j.thromres.2020.03.003, PMID: 32192995 PMC8780211

[71] Moore KL Andreoli SP Esmon NL Esmon CT Bang NU. Endotoxin enhances tissue factor and suppresses thrombomodulin expression of human vascular endothelium in vitro. J Clin Invest. (1987) 79:124–30. doi: 10.1172/JCI112772, PMID: 3025256 PMC424004

[72] Whitfield C Trent MS. Biosynthesis and export of bacterial lipopolysaccharides. Annu Rev Biochem. (2014) 83:99–128. doi: 10.1146/annurev-biochem-060713-035600 24580642

[73] Saghazadeh A Rezaei N. Inflammation as a cause of venous thromboembolism. Crit Rev Oncol Hematol. (2016) 99:272–85. doi: 10.1016/j.critrevonc.2016.01.007 26811138

[74] Branchford BR Carpenter SL. The role of inflammation in venous thromboembolism. Front Pediatr. (2018) 6:142. doi: 10.3389/fped.2018.00142, PMID: 29876337 PMC5974100

[75] Holstein K Matysiak A Witt L Sievers B Beckmann L Haddad M. LPS-induced expression and release of monocyte tissue factor in patients with haemophilia. Ann Hematol. (2020) 99:1531–42. doi: 10.1007/s00277-020-04075-6, PMID: 32430703 PMC7316670

[76] Trebicka J Krag A Gansweid S Appenrodt B Schiedermaier P Sauerbruch T. Endotoxin and tumor necrosis factor-receptor levels in portal and hepatic vein of patients with alcoholic liver cirrhosis receiving elective transjugular intrahepatic portosystemic shunt. Eur J Gastroenterol Hepatol. (2011) 23:1218–25. doi: 10.1097/MEG.0b013e32834a75dc, PMID: 21971377

[77] Balmer ML Slack E de Gottardi A Lawson MA Hapfelmeier S Miele L. The liver may act as a firewall mediating mutualism between the host and its gut commensal microbiota. Sci Transl Med. (2014) 6:237ra66. doi: 10.1126/scitranslmed.3008618 24848256

[78] Seki E Schnabl B. Role of innate immunity and the microbiota in liver fibrosis: crosstalk between the liver and gut. J Physiol. (2012) 590:447–58. doi: 10.1113/jphysiol.2011.219691, PMID: 22124143 PMC3379693

[79] Nakamoto N Kanai T. Role of toll-like receptors in immune activation and tolerance in the liver. Front Immunol. (2014) 5:221. doi: 10.3389/fimmu.2014.00221 24904576 PMC4032908

[80] Custodio-Chablé SJ Lezama RA Reyes-Maldonado E. Platelet activation as a trigger factor for inflammation and atherosclerosis. Cir Cir. (2020) 88:233–43. doi: 10.24875/CIRU.19000725, PMID: 32116325

[81] Tsuchida T Friedman SL. Mechanisms of hepatic stellate cell activation. Nat Rev Gastroenterol Hepatol. (2017) 14:397–411. doi: 10.1038/nrgastro.2017.38 28487545

[82] Reinhardt C. The gut microbiota as an influencing factor of arterial thrombosis. Hamostaseologie. (2019) 39:173–9. doi: 10.1055/s-0038-1675357, PMID: 30458552

[83] Jäckel S Kiouptsi K Lillich M Hendrikx T Khandagale A Kollar B. Gut microbiota regulate hepatic von Willebrand factor synthesis and arterial thrombus formation via toll-like receptor-2. Blood. (2017) 130:542–53. doi: 10.1182/blood-2016-11-754416, PMID: 28572286

[84] Shin HS Xu F Bagchi A Herrup E Prakash A Valentine C. Bacterial lipoprotein TLR2 agonists broadly modulate endothelial function and coagulation pathways *in vitro* and *in vivo*. J Immunol. (2011) 186:1119–30. doi: 10.4049/jimmunol.1001647, PMID: 21169547 PMC3482611

[85] Cognasse F Hamzeh H Chavarin P Acquart S Genin C Garraud O. Evidence of toll-like receptor molecules on human platelets. Immunol Cell Biol. (2005) 83:196–8. doi: 10.1111/j.1440-1711.2005.01314.x, PMID: 15748217

[86] Blair P Rex S Vitseva O Beaulieu L Tanriverdi K Chakrabarti S. Stimulation of toll-like receptor 2 in human platelets induces a thromboinflammatory response through activation of phosphoinositide 3-kinase. Circ Res. (2009) 104:346–54. doi: 10.1161/CIRCRESAHA.108.185785, PMID: 19106411 PMC2732983

[87] Rex S Beaulieu LM Perlman DH Vitseva O Blair PS McComb ME. Immune versus thrombotic stimulation of platelets differentially regulates signalling pathways, intracellular protein–protein interactions, and alpha-granule release. Thromb Haemost. (2009) 102:97–110. doi: 10.1160/TH08-08-0513, PMID: 19572074 PMC2774228

[88] Duttaroy AK. Role of gut microbiota and their metabolites on atherosclerosis, hypertension and human blood platelet function: a review. Nutrients. (2021) 13:13. doi: 10.3390/nu13010144 PMC7824497 33401598

[89] Engelmann B Massberg S. Thrombosis as an intravascular effector of innate immunity. Nat Rev Immunol. (2013) 13:34–45. doi: 10.1038/nri3345, PMID: 23222502

[90] Kimball AS Obi AT Diaz JA Henke PK. The emerging role of NETs in venous thrombosis and immunothrombosis. Front Immunol. (2016) 7:236. doi: 10.3389/fimmu.2016.00236 27446071 PMC4921471

[91] Brill A Fuchs TA Savchenko AS Thomas GM Martinod K De Meyer SF. Neutrophil extracellular traps promote deep vein thrombosis in mice. J Thromb Haemost. (2012) 10:136–44. doi: 10.1111/j.1538-7836.2011.04544.x, PMID: 22044575 PMC3319651

[92] Seo JD Gu JY Jung HS Kim YJ Kim HK. Contact system activation and neutrophil extracellular trap markers: risk factors for portal vein thrombosis in patients with hepatocellular carcinoma. Clin Appl Thromb Hemost. (2019) 25:107602961882531. doi: 10.1177/1076029618825310 PMC6715110 30808222

[93] Wang Y Luo L Braun OÖ Westman J Madhi R Herwald H. Neutrophil extracellular trap-microparticle complexes enhance thrombin generation via the intrinsic pathway of coagulation in mice. Sci Rep. (2018) 8:4020. doi: 10.1038/s41598-018-22156-5, PMID: 29507382 PMC5838234

[94] Semeraro F Ammollo CT Morrissey JH Dale GL Friese P Esmon NL. Extracellular histones promote thrombin generation through platelet-dependent mechanisms: involvement of platelet TLR2 and TLR4. Blood. (2011) 118:1952–61. doi: 10.1182/blood-2011-03-343061 21673343 PMC3158722

[95] McDonald D Ackermann G Khailova L Baird C Heyland D Kozar R. Extreme Dysbiosis of the microbiome in critical illness. mSphere. (2016) 1:e00199. doi: 10.1128/mSphere.00199-16, PMID: 27602409 PMC5007431

[96] Zhu Y Li Q Jiang H. Gut microbiota in atherosclerosis: focus on trimethylamine N-oxide. APMIS. (2020) 128:353–66. doi: 10.1111/apm.13038, PMID: 32108960 PMC7318354

[97] Tilg H. A gut feeling about thrombosis. N Engl J Med. (2016) 374:2494–6. doi: 10.1056/NEJMcibr1604458, PMID: 27332910

[98] Zhu W Gregory JC Org E Buffa JA Gupta N Wang Z. Gut microbial metabolite TMAO enhances platelet hyperreactivity and thrombosis risk. Cells. (2016) 165:111–24. doi: 10.1016/j.cell.2016.02.011, PMID: 26972052 PMC4862743

[99] Tripodi A Primignani M Chantarangkul V Dell’Era A Clerici M de Franchis R. An imbalance of pro- vs. anti-coagulation factors in plasma from patients with cirrhosis. Gastroenterology. (2009) 137:2105–11. doi: 10.1053/j.gastro.2009.08.045, PMID: 19706293

[100] Basili S Carnevale R Nocella C Bartimoccia S Raparelli V Talerico G. Serum albumin is inversely associated with portal vein thrombosis in cirrhosis. Hepatol Commun. (2019) 3:504–12. doi: 10.1002/hep4.1317, PMID: 30976741 PMC6442692

[101] Wang T Xu J Fu L Li L. Hypertriglyceridemia is associated with platelet hyperactivation in metabolic syndrome patients. Int J Clin Pract. (2020) 74:e13508. doi: 10.1111/ijcp.13508 32279396

[102] Ciavarella A Gnocchi D Custodero C Lenato GM Fiore G Sabbà C. Translational insight into prothrombotic state and hypercoagulation in nonalcoholic fatty liver disease. Thromb Res. (2021) 198:139–50. doi: 10.1016/j.thromres.2020.12.002 33340925

[103] Stine JG Shah NL Argo CK Pelletier SJ Caldwell SH Northup PG. Increased risk of portal vein thrombosis in patients with cirrhosis due to nonalcoholic steatohepatitis. Liver Transpl. (2015) 21:1016–21. doi: 10.1002/lt.24134, PMID: 25845711 PMC6615024

[104] Balan G Trifan A Botezatu D Anton C. Spontaneous bacterial peritonitis: a severe complication of liver cirrhosis. Rev Med Chir Soc Med Nat Iasi. (2011) 115:38–44. PMID: 21688558

[105] Song DS. Spontaneous bacterial peritonitis. Korean J Gastroenterol. (2018) 72:56–63. doi: 10.4166/kjg.2018.72.2.56 30145857

[106] Llovet JM Bartolí R March F Planas R Viñado B Cabré E. Translocated intestinal bacteria cause spontaneous bacterial peritonitis in cirrhotic rats: molecular epidemiologic evidence. J Hepatol. (1998) 28:307–13. doi: 10.1016/0168-8278(88)80018-7 9580278

[107] Papp M Farkas A Udvardy M Tornai I. Bacterial infections in cirrhosis. Orv Hetil. (2007) 148:387–95. doi: 10.1556/oh.2007.27882 17344166

[108] Kim JH Jeon YD Jung IY Ahn MY Ahn HW Ahn JY. Predictive factors of spontaneous bacterial peritonitis caused by Gram-positive bacteria in patients with cirrhosis. Medicine. (2016) 95:e3489. doi: 10.1097/MD.0000000000003489, PMID: 27124049 PMC4998712

[109] Bajaj JS Vargas HE Reddy KR Lai JC O’Leary JG Tandon P. Association between intestinal microbiota collected at hospital admission and outcomes of patients with cirrhosis. Clin Gastroenterol Hepatol. (2019) 17:756–765.e3. doi: 10.1016/j.cgh.2018.07.022, PMID: 30036646

[110] Singh A Cresci GA Kirby DF. Proton pump inhibitors: risks and rewards and emerging consequences to the gut microbiome. Nutr Clin Pract. (2018) 33:614–24. doi: 10.1002/ncp.10181, PMID: 30071147

[111] Tranah TH Edwards LA Schnabl B Shawcross DL. Targeting the gut-liver-immune axis to treat cirrhosis. Gut. (2021) 70:982–94. doi: 10.1136/gutjnl-2020-320786, PMID: 33060124

[112] Wang YD Chen WD Moore DD Huang W. FXR: a metabolic regulator and cell protector. Cell Res. (2008) 18:1087–95. doi: 10.1038/cr.2008.289, PMID: 18825165

[113] Sorribas M Jakob MO Yilmaz B Li H Stutz D Noser Y. FXR modulates the gut-vascular barrier by regulating the entry sites for bacterial translocation in experimental cirrhosis. J Hepatol. (2019) 71:1126–40. doi: 10.1016/j.jhep.2019.06.017, PMID: 31295531

[114] Hanouneh MA Hanouneh IA Hashash JG Law R Esfeh JM Lopez R. The role of rifaximin in the primary prophylaxis of spontaneous bacterial peritonitis in patients with liver cirrhosis. J Clin Gastroenterol. (2012) 46:709–15. doi: 10.1097/MCG.0b013e3182506dbb, PMID: 22878533

[115] Moreau R Elkrief L Bureau C Perarnau JM Thévenot T Saliba F. Effects of long-term norfloxacin therapy in patients with advanced cirrhosis. Gastroenterology. (2018) 155:1816–1827.e9. doi: 10.1053/j.gastro.2018.08.026, PMID: 30144431

[116] Elfert A Abo Ali L Soliman S Ibrahim S Abd-Elsalam S. Randomized-controlled trial of rifaximin versus norfloxacin for secondary prophylaxis of spontaneous bacterial peritonitis. Eur J Gastroenterol Hepatol. (2016) 28:1450–4. doi: 10.1097/MEG.0000000000000724, PMID: 27512927

[117] Madsen BS Havelund T Krag A. Targeting the gut-liver axis in cirrhosis: antibiotics and non-selective β-blockers. Adv Ther. (2013) 30:659–70. doi: 10.1007/s12325-013-0044-1, PMID: 23881723

[118] Reiberger T Ferlitsch A Payer BA Mandorfer M Heinisch BB Hayden H. Non-selective betablocker therapy decreases intestinal permeability and serum levels of LBP and IL-6 in patients with cirrhosis. J Hepatol. (2013) 58:911–21. doi: 10.1016/j.jhep.2012.12.011, PMID: 23262249

[119] Ahluwalia V Betrapally NS Hylemon PB White MB Gillevet PM Unser AB. Impaired gut-liver-brain axis in patients with cirrhosis. Sci Rep. (2016) 6:26800. doi: 10.1038/srep26800, PMID: 27225869 PMC4880966

[120] Wijdicks EF. Hepatic encephalopathy. N Engl J Med. (2016) 375:1660–70. doi: 10.1056/NEJMra1600561 27783916

[121] Olde Damink SW Deutz NE Dejong CH Soeters PB Jalan R. Interorgan ammonia metabolism in liver failure. Neurochem Int. (2002) 41:177–88. doi: 10.1016/S0197-0186(02)00040-2, PMID: 12020618

[122] Butterworth RF. The liver-brain axis in liver failure: neuroinflammation and encephalopathy. Nat Rev Gastroenterol Hepatol. (2013) 10:522–8. doi: 10.1038/nrgastro.2013.99, PMID: 23817325

[123] Bajaj JS Ridlon JM Hylemon PB Thacker LR Heuman DM Smith S. Linkage of gut microbiome with cognition in hepatic encephalopathy. Am J Physiol Gastrointest Liver Physiol. (2012) 302:G168–75. doi: 10.1152/ajpgi.00190.2011, PMID: 21940902 PMC3345956

[124] Kaji K Takaya H Saikawa S Furukawa M Sato S Kawaratani H. Rifaximin ameliorates hepatic encephalopathy and endotoxemia without affecting the gut microbiome diversity. World J Gastroenterol. (2017) 23:8355–66. doi: 10.3748/wjg.v23.i47.8355, PMID: 29307995 PMC5743506

[125] Bajaj JS Salzman N Acharya C Takei H Kakiyama G Fagan A. Microbial functional change is linked with clinical outcomes after capsular fecal transplant in cirrhosis. JCI Insight. (2019) 4:133410. doi: 10.1172/jci.insight.133410 31751317 PMC6975263

[126] Campion D Giovo I Ponzo P Saracco GM Balzola F Alessandria C. Dietary approach and gut microbiota modulation for chronic hepatic encephalopathy in cirrhosis. World J Hepatol. (2019) 11:489–512. doi: 10.4254/wjh.v11.i6.489, PMID: 31293718 PMC6603507

[127] Jia W Rajani C Kaddurah-Daouk R Li H. Expert insights: the potential role of the gut microbiome-bile acid-brain axis in the development and progression of Alzheimer’s disease and hepatic encephalopathy. Med Res Rev. (2020) 40:1496–507. doi: 10.1002/med.21653, PMID: 31808182

[128] O’Mahony SM Clarke G Borre YE Dinan TG Cryan JF. Serotonin, tryptophan metabolism and the brain-gut-microbiome axis. Behav Brain Res. (2015) 277:32–48. doi: 10.1016/j.bbr.2014.07.027 25078296

[129] Iebba V Guerrieri F Di Gregorio V Levrero M Gagliardi A Santangelo F. Combining amplicon sequencing and metabolomics in cirrhotic patients highlights distinctive microbiota features involved in bacterial translocation, systemic inflammation and hepatic encephalopathy. Sci Rep. (2018) 8:8210. doi: 10.1038/s41598-018-26509-y, PMID: 29844325 PMC5974022

[130] Bosch J Groszmann RJ Shah VH. Evolution in the understanding of the pathophysiological basis of portal hypertension: how changes in paradigm are leading to successful new treatments. J Hepatol. (2015) 62:S121–30. doi: 10.1016/j.jhep.2015.01.003, PMID: 25920081 PMC4519833

[131] Arab JP Martin-Mateos RM Shah VH. Gut-liver axis, cirrhosis and portal hypertension: the chicken and the egg. Hepatol Int. (2018) 12:24–33. doi: 10.1007/s12072-017-9798-x, PMID: 28550391 PMC6876989

[132] Trebicka J Reiberger T Laleman W. Gut-liver axis links portal hypertension to acute-on-chronic liver failure. Visc Med. (2018) 34:270–5. doi: 10.1159/000490262, PMID: 30345284 PMC6189544

[133] Simbrunner B Mandorfer M Trauner M Reiberger T. Gut-liver axis signaling in portal hypertension. World J Gastroenterol. (2019) 25:5897–917. doi: 10.3748/wjg.v25.i39.5897, PMID: 31660028 PMC6815800

[134] Steib CJ Hartmann AC Hesler C Benesic A Hennenberg M Bilzer M. Intraperitoneal LPS amplifies portal hypertension in rat liver fibrosis. Lab Investig (2010) 90:1024–1032. doi: 10.1038/labinvest.2010.60, PMID: 20212458

[135] Villanueva C Albillos A Genescà J Abraldes JG Calleja JL Aracil C. Development of hyperdynamic circulation and response to β-blockers in compensated cirrhosis with portal hypertension. Hepatology. (2016) 63:197–206. doi: 10.1002/hep.28264, PMID: 26422126

[136] Reiberger T Mandorfer M. Beta adrenergic blockade and decompensated cirrhosis. J Hepatol. (2017) 66:849–59. doi: 10.1016/j.jhep.2016.11.001 27864004

[137] Schwabl P Hambruch E Seeland BA Hayden H Wagner M Garnys L. The FXR agonist PX20606 ameliorates portal hypertension by targeting vascular remodelling and sinusoidal dysfunction. J Hepatol. (2017) 66:724–33. doi: 10.1016/j.jhep.2016.12.005, PMID: 27993716

[138] Zhu Q Zou L Jagavelu K Simonetto DA Huebert RC Jiang ZD. Intestinal decontamination inhibits TLR4 dependent fibronectin-mediated cross-talk between stellate cells and endothelial cells in liver fibrosis in mice. J Hepatol. (2012) 56:893–9. doi: 10.1016/j.jhep.2011.11.013, PMID: 22173161 PMC3307873

[139] Francoz C Durand F Kahn JA Genyk YS Nadim MK. Hepatorenal syndrome. Clin J Am Soc Nephrol. (2019) 14:774–81. doi: 10.2215/CJN.12451018, PMID: 30996046 PMC6500947

[140] Amin AA Alabsawy EI Jalan R Davenport A. Epidemiology, pathophysiology, and management of hepatorenal syndrome. Semin Nephrol. (2019) 39:17–30. doi: 10.1016/j.semnephrol.2018.10.002, PMID: 30606404

[141] Adebayo D Neong SF Wong F. Ascites and hepatorenal syndrome. Clin Liver Dis. (2019) 23:659–82. doi: 10.1016/j.cld.2019.06.002, PMID: 31563217

[142] Huang LT Hung JF Chen CC Hsieh CS Yu HR Hsu CN. Endotoxemia exacerbates kidney injury and increases asymmetric dimethylarginine in young bile duct-ligated rats. Shock. (2012) 37:441–8. doi: 10.1097/SHK.0b013e318244b787 22193869

[143] Peng JL Techasatian W Hato T Liangpunsakul S. Role of endotoxemia in causing renal dysfunction in cirrhosis. J Investig Med. (2020) 68:26–9. doi: 10.1136/jim-2019-001056, PMID: 31324695 PMC8073210

[144] Shah N Dhar D El Zahraa MF Habtesion A Davies NA Jover-Cobos M. Prevention of acute kidney injury in a rodent model of cirrhosis following selective gut decontamination is associated with reduced renal TLR4 expression. J Hepatol. (2012) 56:1047–53. doi: 10.1016/j.jhep.2011.11.024, PMID: 22266601

[145] Kalambokis GN Mouzaki A Rodi M Pappas K Fotopoulos A Xourgia X. Rifaximin improves systemic hemodynamics and renal function in patients with alcohol-related cirrhosis and ascites. Clin Gastroenterol Hepatol. (2012) 10:815–8. doi: 10.1016/j.cgh.2012.02.025, PMID: 22391344

[146] Garcia-Martinez R Noiret L Sen S Mookerjee R Jalan R. Albumin infusion improves renal blood flow autoregulation in patients with acute decompensation of cirrhosis and acute kidney injury. Liver Int. (2015) 35:335–43. doi: 10.1111/liv.12528, PMID: 24620819

[147] Vincent JL De Backer D Wiedermann CJ. Fluid management in sepsis: the potential beneficial effects of albumin. J Crit Care. (2016) 35:161–7. doi: 10.1016/j.jcrc.2016.04.019, PMID: 27481753

[148] Liangpunsakul S Agarwal R. Altered circadian hemodynamic and renal function in cirrhosis. Nephrol Dial Transplant. (2017) 32:333–42. doi: 10.1093/ndt/gfw014 28186574 PMC5837480

[149] Koch DG Fallon MB. Hepatopulmonary syndrome. Clin Liver Dis. (2014) 18:407–20. doi: 10.1016/j.cld.2014.01.003 24679503

[150] Zhang ZJ Yang CQ. Progress in investigating the pathogenesis of hepatopulmonary syndrome. Hepatobiliary Pancreat Dis Int. (2010) 9:355–60. PMID: 20688597

[151] Zhang J Fallon MB. Hepatopulmonary syndrome: update on pathogenesis and clinical features. Nat Rev Gastroenterol Hepatol. (2012) 9:539–49. doi: 10.1038/nrgastro.2012.123, PMID: 22751459 PMC10963041

[152] Tumgor G. Cirrhosis and hepatopulmonary syndrome. World J Gastroenterol. (2014) 20:2586–94. doi: 10.3748/wjg.v20.i10.2586 24627594 PMC3949267

[153] Zhang HY Han DW Zhao ZF Liu MS Wu YJ Chen XM. Multiple pathogenic factor-induced complications of cirrhosis in rats: a new model of hepatopulmonary syndrome with intestinal endotoxemia. World J Gastroenterol. (2007) 13:3500–7. doi: 10.3748/wjg.v13.i25.3500, PMID: 17659698 PMC4146787

[154] Sztrymf B Libert JM Mougeot C Lebrec D Mazmanian M Humbert M. Cirrhotic rats with bacterial translocation have higher incidence and severity of hepatopulmonary syndrome. J Gastroenterol Hepatol. (2005) 20:1538–44. doi: 10.1111/j.1440-1746.2005.03914.x, PMID: 16174071

[155] Gupta S Faughnan ME Lilly L Hutchison S Fowler R Bayoumi AM. Norfloxacin therapy for hepatopulmonary syndrome: a pilot randomized controlled trial. Clin Gastroenterol Hepatol. (2010) 8:1095–8. doi: 10.1016/j.cgh.2010.08.011, PMID: 20816858

[156] Llovet JM Zucman-Rossi J Pikarsky E Sangro B Schwartz M Sherman M. Hepatocellular carcinoma. Nat Rev Dis Primers. (2016) 2:16018. doi: 10.1038/nrdp.2016.18 27158749

[157] Marengo A Rosso C Bugianesi E. Liver cancer: connections with obesity, fatty liver, and cirrhosis. Annu Rev Med. (2016) 67:103–17. doi: 10.1146/annurev-med-090514-013832 26473416

[158] Liu Q Li F Zhuang Y Xu J Wang J Mao X. Alteration in gut microbiota associated with hepatitis B and non-hepatitis virus related hepatocellular carcinoma. Gut Pathog. (2019) 11:1. doi: 10.1186/s13099-018-0281-6, PMID: 30675188 PMC6337822

[159] Nicoletti A Ponziani FR Biolato M Valenza V Marrone G Sganga G. Intestinal permeability in the pathogenesis of liver damage: from non-alcoholic fatty liver disease to liver transplantation. World J Gastroenterol. (2019) 25:4814–34. doi: 10.3748/wjg.v25.i33.4814, PMID: 31543676 PMC6737313

[160] Liu WT Jing YY Gao L Li R Yang X Pan XR. Lipopolysaccharide induces the differentiation of hepatic progenitor cells into myofibroblasts constitutes the hepatocarcinogenesis-associated microenvironment. Cell Death Differ. (2020) 27:85–101. doi: 10.1038/s41418-019-0340-7, PMID: 31065105 PMC7206112

[161] Yu LX Yan HX Liu Q Yang W Wu HP Dong W. Endotoxin accumulation prevents carcinogen-induced apoptosis and promotes liver tumorigenesis in rodents. Hepatology. (2010) 52:1322–33. doi: 10.1002/hep.23845, PMID: 20803560

[162] Ponziani FR Bhoori S Castelli C Putignani L Rivoltini L Del Chierico F. Hepatocellular carcinoma is associated with gut microbiota profile and inflammation in nonalcoholic fatty liver disease. Hepatology. (2019) 69:107–20. doi: 10.1002/hep.30036 29665135

[163] Piñero F Vazquez M Baré P Rohr C Mendizabal M Sciara M. A different gut microbiome linked to inflammation found in cirrhotic patients with and without hepatocellular carcinoma. Ann Hepatol. (2019) 18:480–7. doi: 10.1016/j.aohep.2018.10.003, PMID: 31023615

[164] Duan Y Llorente C Lang S Brandl K Chu H Jiang L. Bacteriophage targeting of gut bacterium attenuates alcoholic liver disease. Nature. (2019) 575:505–11. doi: 10.1038/s41586-019-1742-x, PMID: 31723265 PMC6872939

[165] Tili E Michaille JJ Piurowski V Rigot B Croce CM. MicroRNAs in intestinal barrier function, inflammatory bowel disease and related cancers-their effects and therapeutic potentials. Curr Opin Pharmacol. (2017) 37:142–50. doi: 10.1016/j.coph.2017.10.010, PMID: 29154194 PMC5938753

[166] Macnaughtan J Ranchal I Soeda J Sawhney R Oben J Davies N. Oral therapy with non-absorbable carbons of controlled porosity (YAQ-001) selectively modulates stool microbiome and its function and this is associated with restoration of immune function and inflammasome activation. J Hepatol. (2015) 62:S240–13. doi: 10.1016/S0168-8278(15)30110-0

